# Developing Integrated Clinical Pathways for the Management of Clinically Severe Adult Obesity: a Critique of NHS England Policy

**DOI:** 10.1007/s13679-020-00416-8

**Published:** 2020-11-12

**Authors:** Jonathan M. Hazlehurst, Jennifer Logue, Helen M. Parretti, Sally Abbott, Adrian Brown, Dimitri J. Pournaras, Abd A. Tahrani

**Affiliations:** 1 Institute of Metabolism and Systems Research, The Medical School, University of Birmingham, Edgbaston, Birmingham, B15 2TT UK; 2 Centre for Endocrinology, Diabetes and Metabolism, Birmingham Health Partners, Birmingham, UK; 3 Department of Diabetes and Endocrinology, University Hospitals Birmingham NHS Foundation Trust, Birmingham, UK; 4 Lancaster Medical School, Lancaster University, Lancaster, UK; 5 Norwich Medical School, University of East Anglia, Norwich, UK; 6 Department of Bariatric Surgery, University Hospitals Birmingham NHS Foundation Trust, Birmingham, UK; 7 Centre for Obesity Research, University College London, London, UK; 8 National Institute of Health Research, UCLH Biomedical Research Centre, London, UK; 9 Department of Upper GI Surgery, Southmead Hospital, Bristol, UK; 10 Bristol Weight Management and Bariatric Service, Southmead Hospital, Bristol, UK

**Keywords:** Obesity management, Obesity, NHS, Clinical pathways, Tier 3, Tier 2, Tier 4, Weight management, Medical management, Integrated pathway

## Abstract

**Purpose of the Review:**

Pathways for obesity prevention and treatment are well documented, yet the prevalence of obesity is rising, and access to treatment (including bariatric surgery) is limited. This review seeks to assess the current integrated clinical pathway for obesity management in England and determine the major challenges.

**Recent Findings:**

Evidence for tier 2 (community-based lifestyle intervention) and tier 3 (specialist weight management services) is limited, and how it facilitates care and improve outcomes in tier 4 remains uncertain. Treatment access, rigidity in pathways, uncertain treatment outcomes and weight stigma seems to be major barriers to improved care.

**Summary:**

More emphasis must be placed on access to effective treatments, treatment flexibility, addressing stigma and ensuring treatment efficacy including long-term health outcomes. Prevention and treatment should both receive significant focus though should be considered to be largely separate pathways. A simplified system for weight management is needed to allow flexibility and the delivery of personalized care including post-bariatric surgery care for those who need it.

## Introduction

Obesity is a global public health priority. Preventing and treating obesity represents a significant opportunity to improve people’s quality of life and the health of society and reduce the financial pressures on the National Health Service (NHS). Obesity prevalence in England has increased from 14.9% in 1993 to 26.9% in 2015 [1], and it is projected that an additional 11 million people will be living with obesity in the UK by 2050 [2]. In 2015 the NHS spent £6.1 billion on overweight- and obesity-related ill health, and the cost of obesity to the wider society was estimated to be £37 billion [1]. The cost is projected to increase to just under £50 billion in 2050 if obesity rates rise as predicted [3]. Therefore, it is no surprise that the NHS, Public Health England and the wider healthcare system are highly interested in both the prevention and treatment of obesity though this has not translated into clear and effective pathways for either prevention or treatment.

In 2011, the Department of Health published a policy paper “Healthy Lives, Healthy People: A Call to Action on Obesity in England”, which sets a target to reduce the prevalence of obesity in adults by 2020 by providing strategies for the prevention and treatment of obesity [4]. Despite this, the prevalence of obesity has continued to increase, yet the provision of weight management services and obesity treatment in the UK remains variable geographically and relatively limited in comparison to other European countries, most of which have lower prevalence of obesity [5–7]. It appears that the approaches taken to date have not been effective in treating many patients with obesity. In addition, this is also despite multiple documents and policies being issued by the National Institute for Health and Care Excellence (NICE) (clinical guidelines 43 and 189; quality standards 127; public health guidelines 42, 46, 47 and 53 [8–15]; from Public Health England (Health matters: obesity and the food environment, Joined up clinical pathways for obesity) and NHS England (Five Year Forward View, Healthy Lives, Healthy People: A Call to Action on Obesity in England), amongst others [1, 16].

Therefore, for the reasons listed above, it is our view that the NHS did not get the integrated pathway for weight management right. However, that does not mean that there are not positive elements within the system. To justify this opinion, we will describe the current weight management tiered system, its origins and purpose and assess its outcomes. We will also describe the challenges faced by the integrated clinical pathways.

## The Integrated Clinical Pathways for the Management of People with Severe Obesity: Structure and Purpose

**Fig. 1:**
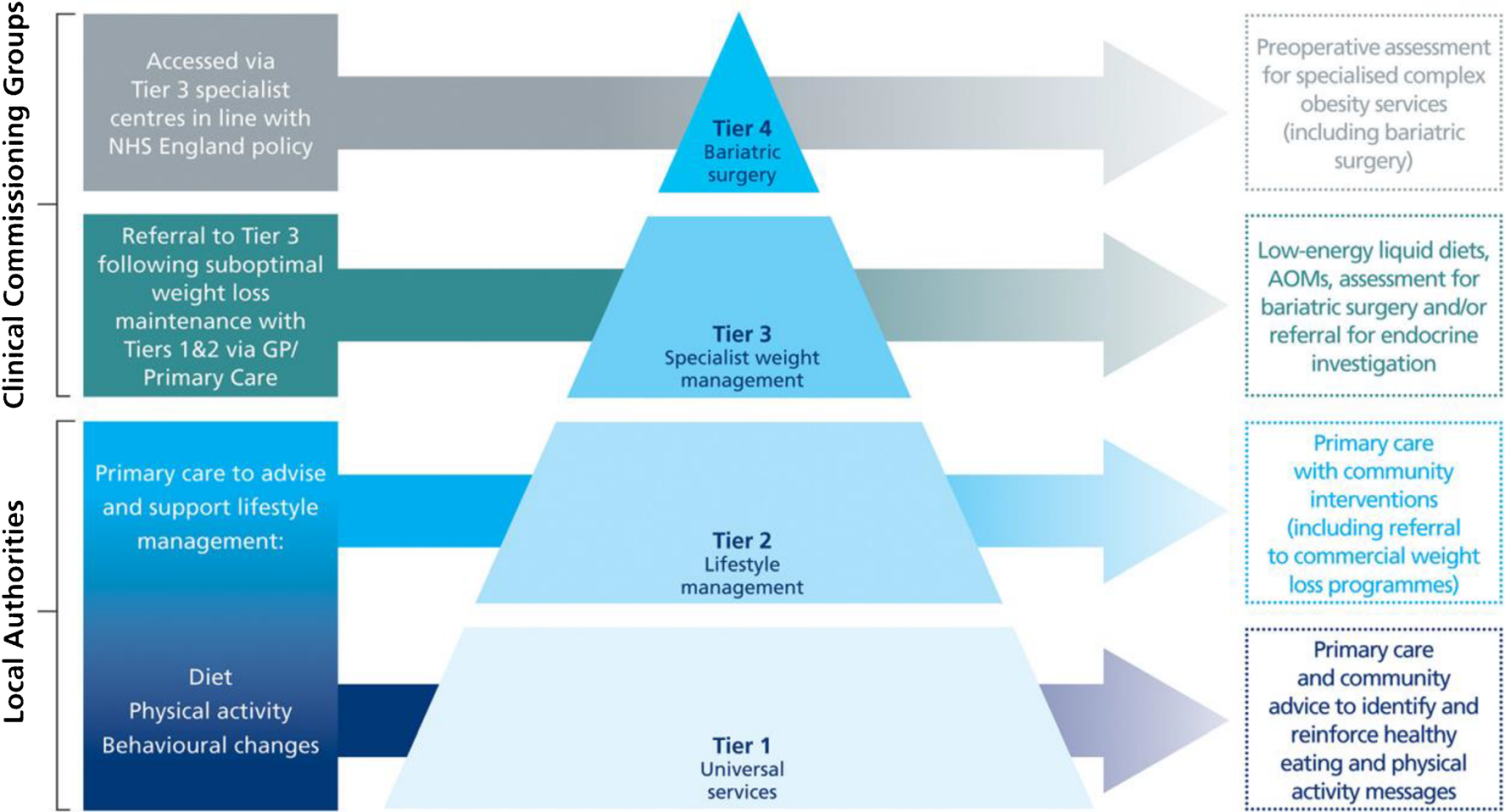
The tiered weight management system in England. Adapted from Wilding 2018 [19]

Within the NHS in England, current obesity management is delivered through a tiered system [17] (Table 1). In 2006, NICE Clinical Guidance (CG) 43 recommended that people with severe obesity should be treated in a specialist setting that has the necessary infrastructure (including equipment) and expertise [12]. NICE CG 43 also recommended that the shared decision-making process between the clinical team and the patient should be adopted and that the treatment plan should be personalized and adapted to the person’s preferences, initial fitness, health status and lifestyle [12]. Seven years later, in 2013, the Action on Obesity report from the Royal College of Physicians concluded that the multidisciplinary services necessary to manage patients with obesity and its complications were poorly developed within the UK and that the response of the NHS to obesity was highly variable [18]. In 2014, the Department of Health Working Group report on the joined up clinical pathways for obesity described the 4 tiers (Fig. 1). Tier 1 is a universal intervention aimed at prevention and re-enforcement of healthy lifestyle principles. Tier 2 is lifestyle and weight management services that can include commercial weight management providers, and it is often time limited to 12 weeks and typically consists of group sessions covering diet, physical activity and behavioural change. Tier 3 is clinician-led weight management services that consist of a multidisciplinary team (MDT) including specialist dietitians, nurses, psychologists and physiotherapists that could be delivered in either primary or secondary care. Tier 4 is bariatric surgery with MDT support pre- and post-surgery (Table 1) [16].

**Table 1:** Summary of the tiered weight management system in England

Tiers	Description	Location	Commissioning lead (primary responsibility agency)	Referral criteria	Patient journey—what are the characteristics of
1 Behavioural	Universal interventions (prevention and reinforcement of healthy eating and physical activity messages). Includes public health and national campaigns. Brief advice	Various	Local authorities responsible for the provision of community-based interventions which encourage healthy eating and physical activity		Overweight. Exit to either tier 2 or exit from pathway
2 Weight management services	Lifestyle weight management services. Normally time limited	Community/GP practice	Local authorities responsible for commissioning lifestyle weight management services. Local authorities as lead agency engaging CCG’s and NHS	Locally determined	Individual defined as having overweight and needs personal directed intervention/s in the community. Entry either self-referred or referred, possibly from tier 1. Exit from pathway. Continuation with tier 2 services. Exit to tier 3
3 Clinician led multidisciplinary team (MDT).	An MDT clinically led team approach, potentially including physician (including consultant or GP with a special interest), specialist nurse, specialist dietitian, psychologist, psychiatrist and physiotherapist	Location flexible—hub/community/GP practice/secondary care setting	CCGs as the future primary commissioners for tier 3 services, engaging with LA and NHS	BMI ≥ 35 kg/m^2^ with co-morbidities or ≥ 40	A person with obesity with complex needs who has not responded to previous tier interventions. Engagement in tier 3 does not automatically lead to surgery. Entry from either tier 2 or tier 4 or direct entry. Exit to either tier 2 or tier 4 or exit from pathway
4 Surgical and non-surgical	Bariatric surgery, supported by MDT pre- and post-op		NHS England is responsible for the assessment and provision of surgery in the short term. In recognizing the benefits of integrated commissioning, NHS England to conduct an early consideration of the elements of tier 4 that should transfer to CCG commissioning in the medium term	BMI ≥ 35 kg/m^2^ with co-morbidities or ≥ 40	Entry must have engaged with tier 3. Exit to tier 3 (post-op support)

*CCG* clinical commissioning groups. Tier 4 is currently funded by the CCGs not NHS England. At the time of the writing of this table, bariatric surgery was funded by NHS England. Adapted from [16]

However, the Department of Health also stated that “… these definitions represent the considered views of the majority of the group at the time and were used as a reference to understand the context of Tier 3 and 4. They are provided for information rather than as a definition” [16]. In addition, the definitions regarding which patient population should receive which tier of care lack clarity. Taken together, it is therefore unsurprising that provision of weight management services remains inconsistent and there is heterogeneity in provision. However, NICE has provided clear guidance and recommendations on when to consider referral to tier 3 services [14], which include the following:

1. The underlying causes of living with overweight or obesity need to be assessed.
2. The person has complex disease states or needs that cannot be managed adequately in tier 2 (e.g. the additional support needs of people with learning disabilities).
3. Conventional treatment has been unsuccessful.
4. Drug treatment is being considered for a person with a body mass index (BMI) of more than 50 kg/m^2^.
5. Specialist interventions (such as a very-low-calorie diet) may be needed.
6. Bariatric surgery is being considered.

In addition, NICE CG 189 recommended that bariatric surgery should be considered if the patient fulfils all of the following criteria:

1. BMI of 40 kg/m^2^ or more, or between 35 and 40 kg/m^2^ and other significant disease (e.g. type 2 diabetes or high blood pressure) that could be improved with weight loss.
2. All appropriate non-surgical measures have been tried, but the person has not achieved or maintained adequate, clinically beneficial weight loss.
3. The person has been receiving or will receive intensive management in a tier 3 service.
4. The person is generally fit for anaesthesia and surgery.
5. The person commits to the need for long-term follow-up.

NICE CG 189 also stated that bariatric surgery is the treatment of choice for adults with a BMI ≥ 50 kg/m^2^ when other interventions have not been effective and that surgery should be performed by an experienced surgeon in specialist centres with access to a multidisciplinary team [12]. Patient selection processes should ensure that only those patients who stand to benefit the most from surgery are referred. This policy mandated that patients should have received and complied with a tier 3 weight management service for 12–24 months prior to referral to bariatric surgery except for patients with BMI > 50 kg/m^2^ where 6 months in tier 3 were deemed adequate. This commissioning policy defined the MDT in tier 3 to be led by a professional with a specialist interest in obesity and should include a physician, specialist dietitian, nurse, psychologist and physical exercise therapist, all of whom must also have a specialist interest in obesity [17]. The role of the MDT before considering bariatric surgery was defined as “assessment of evidence that all suitable non-invasive options have been explored and trialled and individualised patient focus and targets”, to provide education regarding diet and physical activity, exclude underlying endocrine conditions and evaluate and manage comorbidities and psychological factors relevant to obesity. In addition, the policy stated that tier 3 should evaluate patients’ engagement in non-surgical tier 3/4 services. It was suggested that “Engagement can be judged by attendance records and achievement of pre-set individualised targets, for example steady and sustained weight loss of 5-10% or maintaining constant weight whilst stopping smoking” [17]. The policy was recommended referring for bariatric surgery patients who have been unable to lose clinically significant weight (i.e. enough to modify comorbidities) during the period of intervention.

Overall, based on these guidelines, the main objectives of tier 3 weight management services have been to achieve clinically meaningful weight loss in patients with obesity-associated comorbidities in those not considering surgery. In those who want (and are eligible for) bariatric surgery, tier 3 would ensure that all other treatment options have been tried and failed and “select” those who are likely to do better after surgery with suggested ways of patient selection through attendance to tier 3 and achievement of 5–10% weight loss, although pre-surgery weight loss is not a funding requirement for bariatric surgery.

So below we consider the evidence whether tiers 2 and 3 can deliver and are delivering these recommended roles.

## Tier 2 Weight Management Services: What Is the Evidence?

Tier 2 weight management services, which are community based, have been shown to be effective in the case of utilizing commercial weight management programmes. Referral from primary care to commercial weight management programmes resulted in a modest, but significant weight loss compared to the standard care over a 12-month period in a multinational RCT [− 5.06 kg (SE 0.31) vs. − 2.25 kg (0.21), adjusted difference – 2.77 kg, 95% CI − 3.50 to − 2.03] with the last observation carried forward; but the trial had a high dropout rate of 55% [20]. In another RCT primary care, physicians randomly assigned participants to one of two 30-s interventions: advice regarding weight loss vs. referral to a weight management group (12 sessions of 1 h each, once per week). The adjusted difference in weight loss was 1.43 kg (95% CI 0.89–1.97) in favour of the referral intervention [21]. Overall, these programmes result in a modest but meaningful weight loss at 12 months (around 2.2–5 kg on average), and they were superior to general practice or pharmacy-led 1-2-1 counselling [22–25]. In addition, these programmes have been shown to have modest effects on other health outcomes such as glycaemic control, blood pressure and lipids [26, 27].

## Tier 3 Weight Management Services: What Is the Evidence?

### Outcomes of Tier 3 Weight Management Services in England

A clinically meaningful weight loss was defined by NICE as 5–10% as there is evidence that this amount of weight loss can have a favourable impact on obesity comorbidities, although it is acknowledged that a higher percentage weight loss may be needed in people with BMI ≥ 35 kg/m^2^ [28]. There are limited data on the outcomes of tier 3 services in England, such as weight loss and improvements in health, comorbidities and quality of life. Furthermore, there are currently no published data on long-term outcomes following discharge from tier 3. Below is a summary of the studies to date that have assessed weight loss and health outcomes, as well as retention rates in English tier 3 weight management services. Some of these studies are only available as abstracts and therefore have only reported limited data. The available data regarding English tier 3 are spread between community-based [29–32] and secondary care-based services [33–36]. Follow-up duration was between 6 and 24 months. Percentage of participants losing ≥ 5% weight loss on completion of the program varied significantly ranging from 22% [35] to > 70% [30, 36]. The attrition rate varied significantly and was not necessary a feature of follow-up duration [29, 35] (Table 2).

**Table 2:** Summary of the available tier 3 data in England

Reference	Location	Sample size	Follow-up (months)	% losing ≥ 5% who completed follow-up	% lost to the service by the end of follow-up
Senior 2013 [36]	Rotherham	3325	6	72	51
Jennings 2014 [30]	Fakenham	828	12	72	46
Wright 2015 [29]	Birmingham community	144	12	22 (lost > 10%)	10
Brown 2015 [33]	Birmingham secondary	828	6	32	44
Hughes 2015 [31]	Fakenham	213	12	60	26
Kininmonth 2016 [34]	Wakefield	280	6	16*	32
Nield 2016 [32]	Sheffield	288	6	30	60
Steele 2017 [37]	Liverpool	1249	24	24.1	60
Fountain 2019 [35]	Derby	430	6	22	78

*Intention to treat analysis available rather than data relating to those who had completed the program

In a systematic review tier 3 or UK pre-bariatric weight management services, which included low energy diets, 43.4% and 29.4% achieved ≥ 5% and 10% weight loss respectively [38]. This was associated with improved metabolic health outcomes, but the studies were overall of poor quality with high risk of bias [38]. In another more recent systematic review that examined 20 studies including low energy diets (LED) and studies in tiers 2 and 3 weight management as well as studies from before, the tiered system showed high dropouts with modest weight losses in the studies not using LED [39]. Another systematic review of 14 studies of multicomponent interventions in the UK or Ireland (including LED and VLED) showed weight losses between 2.2 and 12.4 kg [40]. But when the VLED and LED were excluded, the weight loss was modest between 2 and 6 kg. The dropouts were high (43–62%), and achievement of ≥ 5% weight loss amongst completers ranged from 32 to 51% [40].

The above-described results of tier 3 services are consistent with what can be achieved with lifestyle interventions. In a meta-analysis of 32 RCTs that compared weight management programmes to routine care or active control, the weighted mean difference (95%CI) of weight loss between the intervention and the control groups was − 3.75 (− 5.17 to − 2.33) kg and − 2.99 (− 4.64 to − 1.35) kg at 12 and 24 months respectively [41]. The same study found that weight management programmes were cost-effective [41]. The NICE public health guideline PH53 in 2014 considered 29 randomized controlled trials of lifestyle weight management programmes lasting 12 months or longer; 7 reported outcomes at ≥ 3 years, and none reported outcomes beyond 5 years [9]. The guideline stated that “Modelling showed that even a small amount of weight loss is cost effective, but only if it is maintained long term” [9].

In summary, the current data regarding the efficacy of tier 3 weight management services is limited. From the available data, it seems that these services are supporting patients to achieve modest 5% weight loss in 50% of patients over 6–12 months, but this varies between services. In addition, the tier 3 services support about 20–25% of patients to achieve ≥ 10% weight loss. However, there are no data reporting on the sustainability of weight loss or associated health benefits on the long term. This is particularly pertinent given that 80% of patients who lose 5% body weight will regain it over 5 years [42]. In addition, although the retention rates varied considerably in the published data, most were below 55%. Low retention rates, modest weight loss and the lack of long-term data make the cost-effectiveness of tier 3 weight management services uncertain and requiring evaluation. Evaluating the cost-effectiveness of tier 3 services is likely to be challenging given significant variation in the structure of each tier 3 service.

### Selection for Bariatric Surgery

In those who want to have bariatric surgery, roles and functions of the MDT have already been outlined above regarding what is expected from tier 3. We will now address the evidence for these below.

#### Achieving Weight Loss to Reduce the Number of People Pursuing Bariatric Surgery

Patients interested in undergoing bariatric surgery want to attain 38% weight loss on average and would be disappointed if they did not lose 26% [43], so the modest 5% weight loss typically seen in tier 3 is not likely to affect patients’ desire to pursue surgical treatment. In addition, to weight loss, patients expect major psychological and physical improvement after surgery [44]. Hence, it is unlikely that tier 3 services will have a significant impact on reducing the need for bariatric surgery considering the modest weight loss and the lack of evidence regarding major improvement in physical and mental domains. In those losing > 10% weight in tier 3, there is limited evidence about maintenance and long-term health outcomes. This needs to be considered within the evidence showing that most weight loss via non-pharmacological and non-surgical interventions is regained over variable amount of time [42, 45, 46]. A reduction in the number of those pursuing surgery may occur given potential new pharmacotherapy options resulting in 15–25% weight loss over 12 months. However, until then, data are lacking regarding the ability of tier 3 services to reduce referral for bariatric surgery.

#### Selecting the Right Patient: Motivation and Engagement

The concept that motivation is important for selecting patients for bariatric surgery is arguable given that bariatric surgery, especially Roux-en-Y gastric bypass and sleeve gastrectomy, results in significant weight loss, weight loss maintenance and metabolic improvement via multiple complex neurohormonal mechanisms rather than simply dietary restriction [47, 48]. Furthermore, bariatric surgery has been shown to work in animals, whice have no apparent motivation for weight loss [49].

In a study by Dixon et al., the readiness to change (RTC) was measured using the University of Rhode Island Change Assessment in 227 consecutive patients undergoing adjustable gastric banding surgery [50]. The RTC scores were blinded until study completion. There was no significant correlation between RTC score and percentage excess weight loss over 2 years (*r* = 0.047, *p* = 0.5). There was no difference in percentage (%) excess weight loss at 2 years between those under and over the median RTC score [52.9 ± 26.9% vs. 52.2 ± 28.3%, *p* = 0.869] [50]. There was no weight loss difference between highest and lowest quartiles or a nonlinear relationship between weight loss and RTC score, and there was no significant relationship between RCT score and compliance [50]. Furthermore, there was no difference in % excess weight loss at 2 years between those who were in the precontemplation (53.8 ± 25.2%), ready to change (53.8 ± 23.6%) or ambivalent (50.8 ± 26.8) stages before surgery [50].

In another study of 64 patients (mean BMI 51 ± 8 kg/m^2^) undergoing laparoscopic sleeve gastrectomy, % excess weight loss over an average follow-up of 20 months was not correlated with motivation [51]. In this study, motivation was assessed using a motivation survey that was developed with 10 items asking how strongly patients were “self-motivated” to lose weight or motivated by their “social environment” (i.e. partner, family/children, friends, colleagues and employer) or “treatment environment” (i.e. physician, health insurance, nutritionist and therapist) [51]. Patients indicated their answers on a 5-point scale that ranged from 1 = not at all to 5 = very strong [51]. In another study, the weight loss 3 years post-laparoscopic adjustable gastric banding was similar in publicly funded vs. self-funded patients (mean excess weight loss % 59.7 vs. 61.8%, *p* = 0.784, 50% excess weight loss achieved in 55.2 vs. 66.0%, *p* = 0.349), suggesting that motivation by self-funding did not affect post-surgical weight loss [52].

Even in the context of medical weight management, the Dieting Readiness Test (DRT) did not predict program attendance or weight loss in 410 adults with obesity seeking weight management in a tertiary centre [53].

Another important point is that maximum weight loss can be predicted by early post-operative weight loss and the variation in post-surgery weight loss follows a normal distribution which is similar to the treatment effects of surgical or non-surgical interventions for conditions other than obesity [54]. This suggests that this is likely due to biological factors rather than motivation.

Hence, there is currently no clinical evidence that motivation and engagement in before surgery either contribute to or predict better weight loss outcomes after bariatric surgery. However, whether it can predict outcomes other than weight loss or lower adverse events after surgery is currently unknown. In addition, it must be noted that the results of the studies such as the ones described above were dependent on the methods of assessment of motivation or engagement. The notion that motivation is important in predicting bariatric surgery outcomes probably stems from obesity stigma the perception that obesity is a “choice” rather than a chronic disease.

#### Selecting the Right Patient: Achieving 5–10% Weight Loss before Surgery

Whether weight loss during medical weight management predicts post-bariatric surgery weight loss is controversial. In a systematic review that was published in 2012, mandatory pre-operative weight loss was associated with greater post-surgical weight loss (over 3 to 48 months) in 6 studies, no association in 7 studies and negative association in 1 study (N from all studies 3254) [55]. Similar conflicting results were shown in a more recent systematic review in 2014 [56]. In a more recent study from 2018 that included 218 patients, pre-operative weight loss during medical weight management was not an independent predictor of weight loss at 12 months post-bariatric surgery [57]. In a study of 141 patients who underwent laparoscopic sleeve gastrectomy, there was no correlation between pre-op % BMI change and 12 months post-surgery % BMI change and no differences in excess % BMI loss between different amounts of % BMI change before surgery [58].

In a study from Birmingham, UK, which does not mandate weight loss in tier 3 before referring to surgery, and that included 208 patients (LAGB *n* = 128, RYGB *n* = 80), 197 participants (94.7%) and 183 participants (88.0%) attended follow-up at 12 and 24 months respectively [59]. There was no difference in post-operative weight loss at 12 or 24 months between those with ≥ or < 5% weight loss during a tier 3 weight management services. Weight loss in tier 3 did not predict weight loss at 12 or 24 months despite adjustment for age, gender, ethnicity, baseline weight (kg), time in tier 3 and type 2 diabetes. In another study from Birmingham that included 45 patients with type 2 diabetes, there was no significant relationship between the % weight loss induced by glucagon-like peptide 1 (GLP-1) receptor agonists during tier 3 services and 12 months post-surgical (band, sleeve, bypass) % weight loss [60]. Interestingly, the correlation coefficients were negative (especially in the gastric bypass and sleeve gastrectomy group) suggesting higher post-operative weight loss in those with less weight loss following GLP-1 agonist treatment [60].

Overall, there is no convincing evidence that weight loss during medical weight management predicts post-operative weight loss. Data specific to tier 3 services in that regard are limited to the two studies described above, and the quality of that evidence is limited by the observational nature of the studies. However, based on the available data, achieving a mandatory weight loss target in tier 3 as a pre-condition to referral to bariatric surgery is not justified. It should also be noted that bariatric surgery outcomes are not limited to weight loss and whether mandatory weight loss in tier 3 can improve other clinical and metabolic outcomes or quality of life is currently unknown.

The above discussion regarding 5–10% weight loss in tier 3 should not be confused with the pre-operative weight loss achieved by the “liver shrinking” diet (which varies between LED, VLED and ketogenic diet) that has been shown to reduce liver volume before bariatric surgery and might have a favourable impact on wound healing, hospital stay and post-surgery complications [61–64].

#### Improving Post-Surgical Outcomes: Addressing Mental Health and Eating Disorders

Mental health disorders, such as depression, anxiety, body dysphoria, poor self-esteem and eating disorder, are very common in patients with obesity undergoing bariatric surgery [65]. Bariatric surgery has also been reported to be associated with increased risk of self-harm and suicide. In a systematic review, the post-bariatric suicide event rate was 2.7/1000 patients (95% CI 0.0019–0.0038), and the suicide/self-harm attempt event rate was 17/1000 patients (95% CI 0.01–0.03) [66]. The self-harm/suicide attempt risk was higher after vs. before bariatric surgery (OR 1.9 (95% CI 1.23–2.95)), and when compared to age, gender and BMI matched control population (OR 3.8 (95% CI, 2.19–6.59)) [66]. The increased of self-harm occurs following all bariatric procedures although highest post-RYGB [67]. However, whether these mental health disorders predict post-surgical weight loss is unclear, and studies have shown conflicting results. For example, a systematic review in 2012 found that three studies showed positive associations, 13 studies showed neutral associations, and four showed negative associations between binge eating and post-operative weight loss [55]. Similarly, with regard to emotional eating, three studies in this review showed neutral associations, and three studies showed negative associations; and in regard to binge eating, three studies reported that patients with pre-operative binge eating lost more weight post-operatively than those without binge eating, thirteen studies reported no association, and four studies reported a negative association [55]. With regard to depression, one study showed a positive association, fourteen studies showed neutral associations, and four studies showed negative associations, and similar conflicting results were shown in regard to other psychological disorders as well as history of sexual abuse [55]. The recent British Obesity and Metabolic Surgery Society (BOMSS) guidelines regarding pre- and post-surgical psychological weight management also acknowledged the importance of providing post-surgical psychology support to patients with bariatric surgery [65]. Particularly that many mental health and eating disorders may recur or occur de novo post-surgery and that these conditions are associated with less weight loss and adverse outcomes when they are present post bariatric surgery [65]. In addition, it is widely accepted that it is important to address significant mental health disease to ensure that the patients are able to undergo surgery and make informed choices. However, there appears to be a lack of evidence to support or refute whether addressing mental health disorders pre-surgery improve bariatric surgery outcomes as bariatric surgery is consistently associated with post-operative decreases in the prevalence and severity of depression [68]. The delivery of high-quality psychological assessments and treatments is rather challenging due to the lack of enough psychologists. Hence, alternative strategies are needed including upskilling other members of the multidisciplinary team to perform such duties under the supervision of a clinical psychologist and the reliance on some of the mental health services delivered in the community, although these are not weight management specific.

##### Has the NHS in England Got It Right? Our View and the Challenges

Despite the modest efficacy of tier 2 services (detailed above), there is still a lack of data on long-term outcomes, and these programmes typically have high levels of dropouts, and also cost-effectiveness can vary according to the program [23, 69]. However, they are appropriate for a subgroup of patients with overweight or obesity and applicable at a population level where modest weight loss would be meaningful. Nonetheless, these are not widely accessible. Many areas in the UK do not have tier 2 services, and they have an associated cost if privately funded, which is challenging considering that obesity prevalence is higher in people from disadvantaged socio-economic backgrounds which further increase health inequalities.

Tier 3 services, within the current structure, are only accessible for a sub-set of patients with obesity, i.e. those with complex obesity. In patients not requiring or wanting bariatric surgery, tier 3 seems to result in modest, but clinically significant weight loss over a 6–12-month period, although a 10% weight loss which is more meaningful clinically is only achieved in less than 20% of patients as described above. However, the few studies reviewing tier 3 services generally had methodological weaknesses, and cost-effectiveness is unclear due to high dropout rates, the lack of long-term data and the variation in the structure of the programmes. Furthermore, the evidence for impact of tier 3 on outcomes other than weight is very limited, and there is a lack of data in regard to hard outcomes such as mortality or cardiovascular disease. So, in short, for this group of patients, tier 3 appears to be modestly effective, but is it the best way to deliver these benefits? Hence, the current approaches to tier 3 might need to be revisited.

In the context of bariatric surgery, the current tier 3 is almost set up to fail most of its objectives particularly in terms of “selecting” those who are likely to achieve greater weight loss post-surgery and reducing the number of patients needing surgery in a significant manner. It is virtually impossible for tier 3 services to be effective in “selecting” the best candidates for bariatric surgery (in terms of weight loss achieved) due to the lack of evidence to support this selection process and the lack of reliable predictors of outcomes. As a result, whichever criteria used to “select” patients by individual services have become barriers between patients and surgery rather than delivering care and personalizing treatment approaches. In addition, considering the modest weight loss achieved in tier 3, its ability to reduce the need for surgery is likely to be negligible and is based on very simplistic assumptions that do not take into account either the complexity of obesity or bariatric surgery. In addition, it has been shown that patients undergoing bariatric surgery can have unrealistic expectations in regard to weight loss. Therefore, the modest weight loss likely to be achieved in tier 3 will not be satisfactory to the patient in many cases [70].

However, tier 3 services still have an important role to play in preparing patients for surgery in terms of education, managing patient expectations, addressing and optimizing obesity-related complications prior to surgery and helping patients to make informed choices. In addition, it can identify patients who have complex mental health needs that either need addressing before surgery or require close observation and possibly intervention if persisting after surgery [71]. Nonetheless, to perform the above-mentioned care, 12–24 months of medical weight management prior to surgical referral may not be required in most patients, and such duration is not supported by evidence. The current structure of tier 3 is overly rigid with little flexibility to allow a patient-centred approach to be used to meet individualized patient needs. For example, patients who develop post-surgical complex nutritional deficiencies, mental health disorders, difficult to treat metabolic complications such as hypoglycaemia, type 2 diabetes recurrence or weight regain would benefit from access to the tier 3 MDT expertise, but currently tier 3 services do not routinely provide input for patients after surgery [71, 72]. Furthermore, there is currently no data about how to support patients who are discharged from tier 3 back to primary care due to lack of meaningful weight loss, although this could change with the availability of better weight loss pharmacotherapy.

The delivery of integrated weight management pathways faces several challenges. The list below, while not exhaustive, presents some of the main challenges:

1. Access to treatment: The provision of the integrated pathways is not universal, and there is a “postcode lottery” in terms of what is delivered at a regional and local level. In the recent national weight management, mapping exercise geographical coverage of tier 2 was 63%, but the coverage of tier 3 was not possible to identify due the poor response rate from the clinical commissioning groups (CCGs) (18%) [7]. A survey of consultant endocrinologists in 2015 showed an estimated coverage of tier 3 of 60% though more formal mapping was not available (J Wass & K Knight, RCP internal communication). As for bariatric surgery provision, this is very limited in the UK with latest estimates suggest less than 0.002% of the potentially eligible adults have surgery annually [73]. In addition, there are many barriers for referral from primary care to tiers 3 and 4 services even when present, including lack of clear referral criteria, lack of awareness of the services available and their clinical outcomes and funding constraints from the commissioners [74, 75] resulting in only a median (IQR) 3 [1–7]% of patients with BMI ≥ 25 kg/m^2^ referred for weight management intervention in the UK [76]. This lack of service commissioning and referral to specialist services could be in part due to obesity stigma.
2. Overall structure: The current tiered system lacks flexibility. While it might have been designed to deliver different levels of care depending on patient needs, the tiers have become hurdles rather than one continuum. There is a need to have flexibility with patients moving through the tiers depending on their clinical needs and response to treatment. There is also rigidity in the tiered system that makes it difficult to cater for patients’ individual needs; for example, patients who require rapid weight loss to get another procedure (e.g. hip operation) who may end up waiting for prolonged periods of time to access services and may or may not achieve the target weight loss to allow their procedure to be performed. In addition, many patients who would require more intensive input in tiers 3 or 4 end up accessing tier 2 first as a pre-condition to progress in the treatment pathway. All that results in further delay and potentially could set up the individual for multiple cycles of “failed” weight loss attempts which have significant negative impact.
3. Type of patients seen: Although Tier 3 weight management services are supposed to see patients with obesity-related complications, the available data suggest that most patients in tier 3 are free of such complications [35]. This raises a question about how to make sure that the service is accessible to those with greatest clinical need. It appears that the criteria used currently may disadvantage patients with lower BMIs, but established obesity-related complications, who may have significant clinical benefit from weight loss.
4. Lack of long-term outcomes and national registry: Unlike bariatric surgery that has the National Bariatric Surgery Register (NBSR), tier 3 services currently do not have a register. This is complicated further by a lack of an agreed core outcome set to measure in tier 3. However, a recently published study identified core outcomes set for tier 2 services [77]. Many of the contributors to this study also work in tier 3, and many of the outcomes identified in this paper are very reasonable outcomes for a tier 3 service. Recording the same outcomes at a national level for tier 3 would help provide outcome data, including for cost-effectiveness analysis, and potentially identify improved ways to deliver services by learning from those services that are achieving better outcomes.
5. Bariatric surgery and new treatment modalities: Several new pharmacotherapies with significant weight loss (> 10–20%) are in development, and many patients with obesity will be eligible for these medications. However, currently how Tier 3 services can deliver such treatment, how they will be funded, the eligibility criteria and the treatment duration are not clear. In addition, the delivery formula of low energy diets in primary care as per NHS long-term plan [78] remains unclear and currently is being piloted by NHS England [79]. Endoscopic bariatric surgery also is increasingly performed in the private sector but is not widely available in the NHS as yet. In addition, and as described above, only a tiny fraction of people eligible for surgery are able to access it due to multiple factors including lack of funding.
6. High prevalence of obesity: The adult prevalence of obesity and overweight is 64% and for obesity alone (defined as BMI ≥ 30 kg/m^2^) 29% [80]. This Health Survey for England (HSE) data highlighted the increasing prevalence of obesity over the years (1997 18%; 2007 25%; 2017 29%). Obviously, we do not know the exact number of people with obesity who would like to seek weight management services; but it is likely that this number is substantive considering the high prevalence of obesity-related comorbidities and complications. The recent estimates of those who would be eligible for bariatric surgery in England is 3.6 million adults based on applying NICE criteria [12] to HSE 2014 data [73]. Such high demand across all tiers of weight management services is likely to outweigh the capacity of the current integrated pathway, even considering tier 2, which has the largest capacity. In addition, as discussed above, a large proportion of regions do not have access to either tier 2 or tier 3 services.
7. Referral criteria: The current referral criteria to tier 3 weight management services are largely aligned with NICE guidelines regarding referral for bariatric surgery (i.e. BMI ≥ 35 kg/m^2^ with complications or ≥ 40 kg/m^2^ without complications). These criteria deprive many patients who could benefit from tier 3 services from access to the multidisciplinary team and do not take into account individual biological factors such as insulin resistance or beta cell function. Several trials showed that 5–10% weight loss has a favourable impact in patients with obesity-related complications such as pre-diabetes, type 2 diabetes, non-alcoholic fatty liver disease and obstructive sleep apnoea even with a BMI below 35 and in many cases below 30. These patients currently do not have access to tier 3 services.
8. Obesity stigma: It is increasingly recognized that obesity stigma is very common within the healthcare system and is a barrier to healthcare delivery [81, 82]. In 2018, the UK All-Party Parliamentary Group on Obesity reported that only 26% of people with obesity reported being treated with dignity and respect by healthcare professionals when seeking advice or treatment for their obesity, while 42% did not feel comfortable talking to their GP about their obesity [83]. Even experts currently working in obesity demonstrate stigmatizing beliefs [84, 85]. Weight stigma, both external and internal, is harmful to an individual’s psychological health, experiences of healthcare and long-term physical health [86, 87]. Stigma may in part be due to the lack of understanding of the biological causes that drive excess weight amongst healthcare professionals, decision-makers and the public [88]. Hence, there is a need to combat obesity stigma both within and outside the healthcare system. This will require concrete action from policy-makers, improve obesity education in undergraduate curriculums and educate healthcare professionals about obesity and how to avoid stigma. In an attempt to address the latest point, a consensus statement from multiple obesity experts and patients from the UK was issued [89].

Some of these challenges are addressed in the BOMSS and multi-collegiate commissioning guidelines for complex obesity services [90, 91]. These guidelines provide a detailed framework to what should be delivered by primary care and specialist weight management services, the multidisciplinary team structure and quality standards to assess performance. These guidelines still face some challenges including the increased workload of primary care physicians and the training requirements. In addition, increased funding is likely to be required to implement these guidelines, but this could prove challenging due to the lack of clear cost-effectiveness of the current tier 3 services.

## Conclusions

**Fig. 2:**
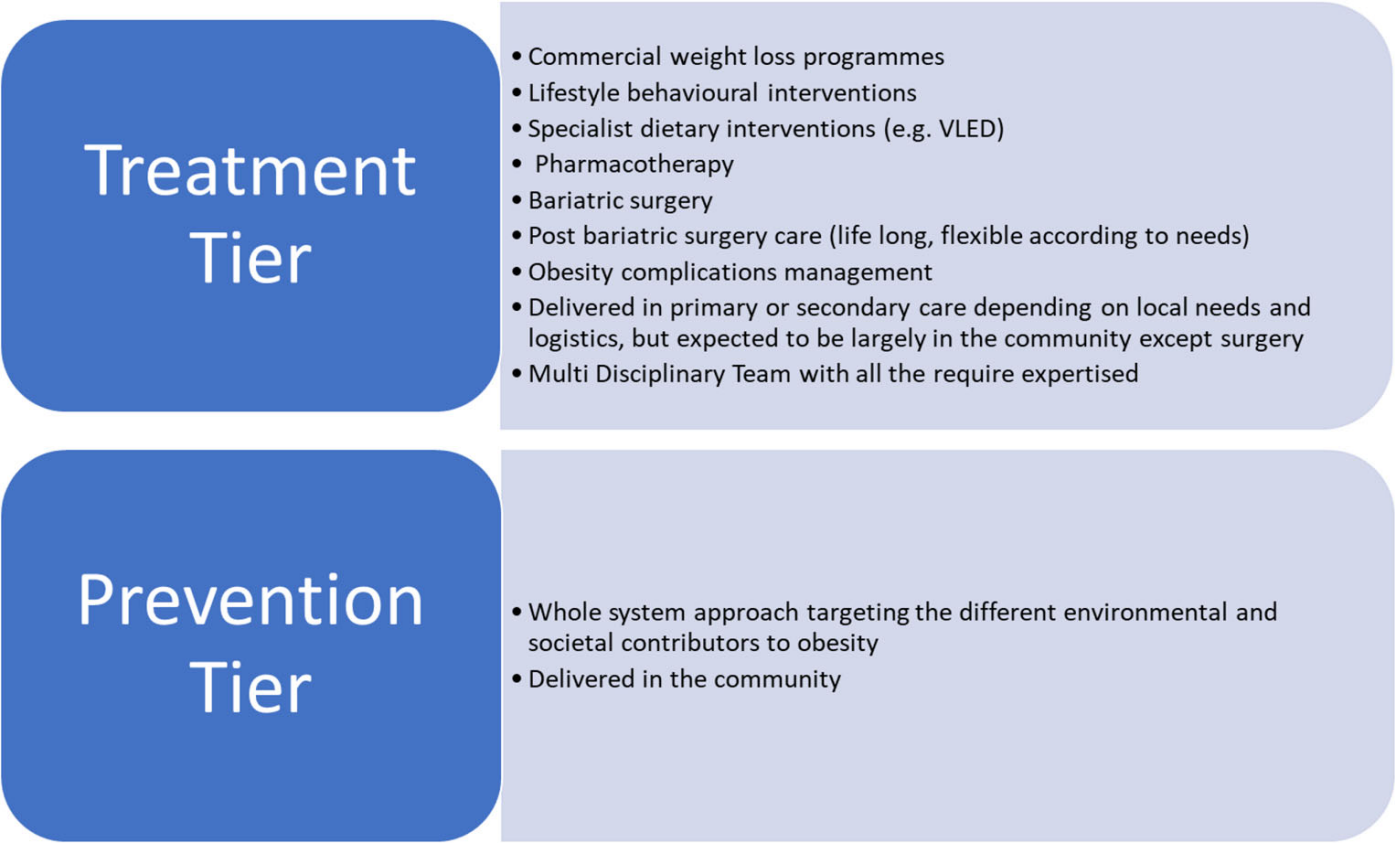
The proposed new weight management system by the authors. *VLED* very low energy diets. The treatment and prevention tier should be delivered simultaneously and in parallel. In the treatment tier, the most suited intervention for the patient needs should be delivered rather than a compulsory step-wise approach. The multidisciplinary team will need to include (but not limited to) clinicians, surgeons, dietitians, physical activity specialists, nurses, appropriate administrative support for the service and data collection for evaluation, and links to all the relevant services in people with obesity (e.g. sleep and liver services)

The integrated tiered weight management pathway was developed in response to the increasing prevalence of obesity in the UK. Tier 1 public health interventions have failed to date to effectively prevent the increasing prevalence of obesity. Tiers 2, 3 and 4 are not available universally across England, and only a fraction of patients who are eligible for these Tiers are referred and seen. Tier 4 (bariatric surgery) is only performed on a very small percentage of patients who are eligible. Tiers 2 and 3 result in modest weight loss. However, their impact on obesity complications is not well studied, and there is a lack of long-term data. Both tiers 2 and 3 suffer from high dropout rates. Tier 3, which is tasked with selecting patients for bariatric surgery, is unable to do this with a high degree of accuracy due to the lack of data available to guide this process. The criteria currently used such as achieving 5–10% weight loss are not evidence based and as such may result in tier 3 being a hurdle to overcome, rather than a facilitator, for bariatric surgery. The current tier 3 system is also rigid and lacks the flexibility to adapt to patient needs. However, as commissioning tier 3 has recently moved to clinical commissioning groups, there is now an opportunity to develop more flexible pathways and move from the previous 12–24 months prior to surgical referral model to one that can deliver the care needed to patients in order to reach bariatric surgery as well as offer support for some patients post bariatric surgery as needed. One possible way to address this lack of flexibility is to have a more integrated but simpler and flexible approach to weight management consistent of two tiers (Fig. 2): a tier for prevention and a tier for treatment. The treatment tier will be flexible to deliver what suits patients’ needs best, and it encompasses the current tiers 2, 3 and 4 allowing easy access to the necessary expertise and a range of treatment option for people with obesity and allowing more involvement of the multidisciplinary team post-bariatric surgery (Fig. 2). Finally, there is a need to train healthcare professionals and address the stigma within the healthcare system.
